# Supplementary material for: Caffeine inhibits Notum activity by binding at the catalytic pocket
Source: Commun Biol. 2020 Oct 8;3:555. doi: 10.1038/s42003-020-01286-5 (PMC7544826; doi:10.1038/s42003-020-01286-5)
Supplement: Supplementary file 1 — Supplementary Information [file 42003_2020_1286_MOESM1_ESM.docx]

**Supplemental Information**

**Caffeine inhibits Notum activity by binding at the catalytic pocket**


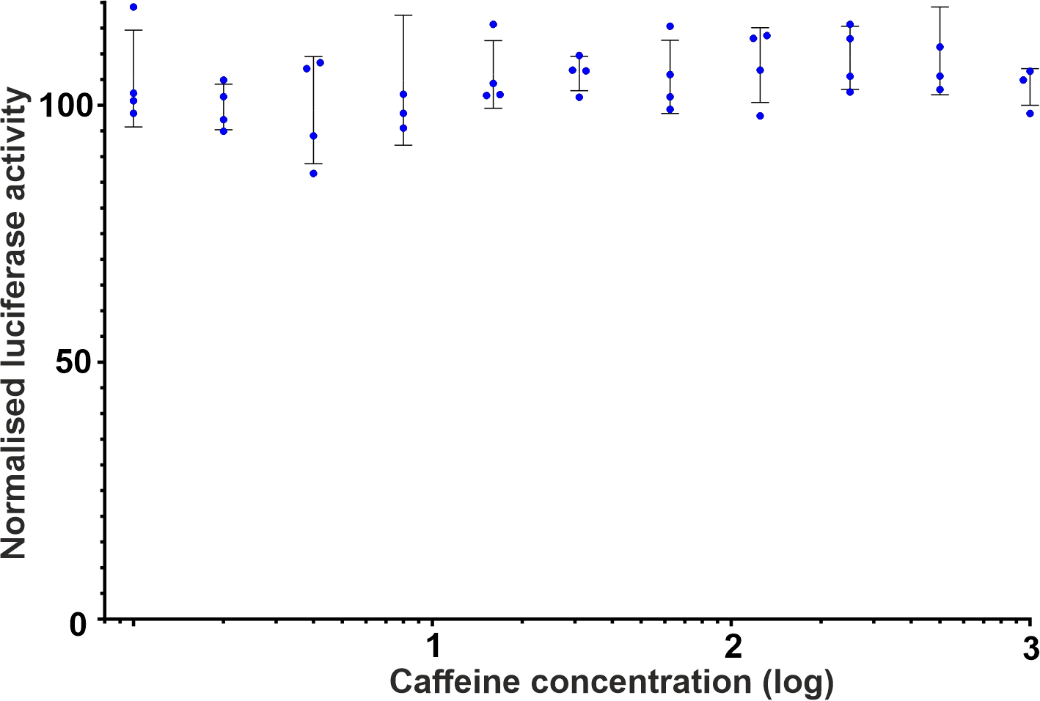


**Supplementary Figure 1.** **Effects of caffeine on Wnt3a induced TOP-Flash luciferase activity.** Normalized luciferase activities from Wnt3a-induced stable STF cell line with caffeine at different concentrations (2-folds dilution series, from 1000 to 1 µM). The average luciferase activities without caffeine were set as 100%.


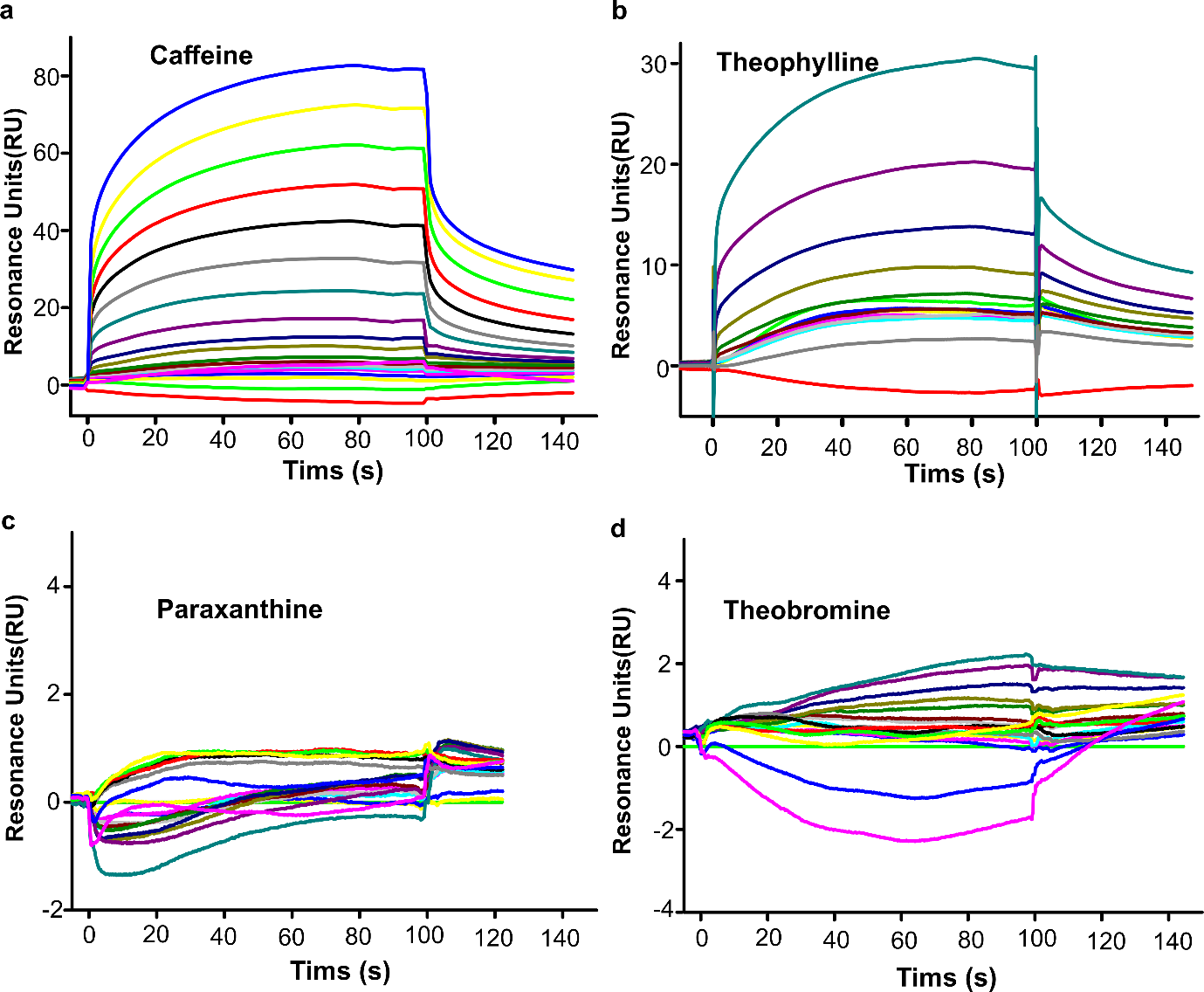


**Supplementary Figure 2.** **The SPR sensorgrams of caffeine and theophylline binding Notum.** Biotinylated Notum was immobilised on a SA chip. Sensograms were recorded with 2-fold dilution series of different ligands: **a** caffeine; **b** theophylline; **c** paraxanthine; **d** theobromine.
