# Supplementary material for: Caffeine inhibits Notum activity by binding at the catalytic pocket
Source: Commun Biol. 2020 Oct 8;3:555. doi: 10.1038/s42003-020-01286-5 (PMC7544826; doi:10.1038/s42003-020-01286-5)
Supplement: Supplementary file 2 — Description of Additional Supplementary Files [file 42003_2020_1286_MOESM2_ESM.pdf]

Description of Additional Supplementary Files

Supplementary Data :

Source data related to Figure1, Figure2 and supplementary Figure 1 and supplementary Figure2.

Supplementary Data 1: Source data related to Figure1a and Figure1b.

Supplementary Data 2: Source data related to Figure 2a.

Supplementary Data 3: Source data related to Figure 2b.

Supplementary Data 4: Source data related to Figure 2c.

Supplementary Data 5: Source data related to Figure 2d.

Supplementary Data 6: Source data related to Supplementary_Figure 1.

Supplementary Data 7: Source data related to Supplementary_Figure 2a.

Supplementary Data 8: Source data related to Supplementary_Figure 2b.

Supplementary Data 9: Source data related to Supplementary_Figure 2c.

Supplementary Data 10: Source data related to Supplementary_Figure 2d.
